# Non-Hermitian quantum walks uncover dynamical quantum phase transitions under self-normal and biorthogonal bases

**DOI:** 10.1038/s41377-025-02069-5

**Published:** 2026-01-04

**Authors:** Guangzhen Li, Luqi Yuan

**Affiliations:** State Key Laboratory of Photonics and Communications, School of Physics and Astronomy, Shanghai Jiao Tong University, Shanghai, China

**Keywords:** Quantum optics, Single photons and quantum effects

## Abstract

The differences in critical times and critical momenta between self-normal and biorthogonal dynamical quantum phase transitions are revealed. The theoretical analysis is experimentally validated through multiple quench processes using a one-dimensional discrete-time non-Hermitian quantum walks.

Non-Hermitian physics has emerged as a rapidly developing frontier field in recent years for breaking through the constraints of Hermitian Hamiltonians in traditional quantum mechanics, providing novel perspectives for investigating exotic physical phenomena in open systems, such as exceptional points, and non-Hermitian topological effects^1–3^. The advances achieved in non-Hermitian physics carry profound theoretical significances in expanding the framework of quantum mechanics and revealing new topological classifications, which also show great potentials in the application fields of high-sensitivity sensing, unidirectional optical devices, topological lasers and quantum computing^4–6^. The research of non-Hermitian physics has recently expanded to non-equilibrium systems, giving rise to the developments of Floquet non-Hermitian physics, non-Hermitian quantum walks and non-Hermitian quantum dynamics^7–9^, which provides crucial foundations for understanding the evolution of open systems and developing new quantum devices. Specifically, non-Hermitian quantum dynamics can unveil unique time evolution behaviors caused by non-Hermitian Hamiltonian in open quantum systems. It breaks through the limitations of traditional closed quantum systems and thus uncovers many counterintuitive dynamic phenomena, for example, the exotic non-Markov memory effects due to the dramatic reconstruction of the system’s eigenstates^10^.

Dynamical quantum phase transitions (DQPTs) are important physical phenomena in non-equilibrium systems^11,12^, which is characterized by the complete vanishing of quantum state overlap between initial and final states during time evolution. Previous researches on DQPTs mostly concentrate on closed Hermitian quantum systems determined by unitary time evolution, while DQPTs in non-Hermitian quantum systems are governed by non-unitary time evolution^13,14^. Therefore, when investigating non-Hermitian DQPTs, the self-normal basis framework^15^ based on Hermitian operators in traditional quantum mechanics faces significant challenges due to the special biorthogonality between the left and right eigenstates, leading to the uncertainty of the time evolution operator. Mathematically, the eigenstates of the Hamiltonian in non-Hermitian systems lose their orthogonality, so it is necessary to consider both left and right eigenstates, which directly brings out the development of biorthogonal basis theory^15^. The separation of left and right eigenstates represents a defining feature distinguishing non-Hermitian systems from Hermitian systems, which also serves as an essential requirement for constructing a complete quantum theoretical framework. More precisely, solutions of the Schrödinger equation in non-Hermitian systems must be expressed in terms of biorthogonal bases to maintain probability conservation, and the time evolution operator e^*-iHt*^ requires expansion in the complete basis formed by both left and right eigenstates. The biorthogonal bases hence emerge as the fundamental mathematical framework for describing the dynamics of open quantum systems. Physically, the introduction of biorthogonal bases offers critical solutions to several challenges in non-Hermitian systems, such as probability conservation, completeness relations, and dynamical descriptions. The non-Hermitian biorthogonal bases not only reconstructs the non-Hermitian topological theory but also establish foundations for parity-time (PT) symmetry quantum mechanics^16,17^, offering new approaches for investigating non-Hermitian quantum dynamics.

The concepts of self-normal and biorthogonality DQPTs under the frameworks of Hermitian and non-Hermitian quantum mechanics respectively have distinct physical origins, while the differences between these two types of DQPTs yet remained unexplored both theoretically and experimentally. A recent research published in Light: Science & Applications led by Prof. Peng Xue from Beijing Computational Science Research Center performed the first comprehensive theoretical and experimental investigation and comparison of self-normal and biorthogonal DQPTs^18^. By selecting different initial states, the researchers thoroughly explore and characterize the key physical quantities of these two types of DQPTs during various quench evolution processes, including Loschmidt rate functions, dynamical topological order parameters, Fisher zeros in the complex plane, and dynamical fixed points. They find that both of these types of DQPTs only appear in the PT-symmetry-unbroken region and disappear in the broken region. Further comparison of quench dynamics between different topological phases reveals that DQPTs only exist between distinct topological phases. The underlying reasons for these phenomena are all related to the fixed points during the evolution process, where DQPTs only occur when two different types of fixed points emerge. Furthermore, they quantify the critical times and critical momenta through analyzing Fisher zeros and fixed points, demonstrating the differences in critical behavior between self-normal and biorthogonal DQPTs. The team finds that the biorthogonal method can better preserve the system’s symmetry, highlighting its unique advantages in non-Hermitian systems. The theoretical predictions are then experimentally validated in one-dimensional discrete-time non-Hermitian quantum walks of single photons (Fig. 1a, b). The experimental platform enables precise parameter adjustment and the introduction of non-unitary dynamics such as gain or loss components, thus exhibiting high controllability and flexibility (Fig. 1c).

**Fig. 1:**
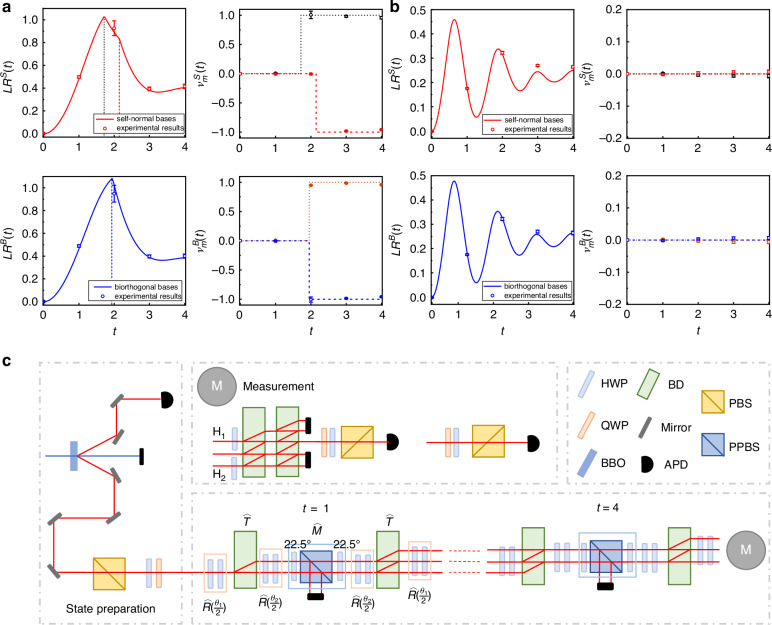
Experimental and theoretical results during different quench processes. **a** The different topological phases in the PT- symmetry-unbroken region. **b** The same topological phases in the PT-symmetry-unbroken region. **c** Experimental setup for the simulation of the self-normal and biorthogonal DQPTs using non-Hermitian QWs. The figure is adapted from ref. ^18^

This research has promoted the development of non-Hermitian quantum physics and DQPTs across multiple dimensions. First, it deepens the understanding of the fundamental characteristics of non-equilibrium dynamics in non-Hermitian systems, establishes a universal theoretical framework for investigating non-Hermitian DQPTs, and lays the foundation for future exploration of more complex and higher-dimensional dynamical topological phenomena, such as non-Hermitian Floquet topological phase transitions and dissipation-induced many-body localization. Secondly, the study reveals a correspondence between two types of quench-induced phase transitions (self-normal and biorthogonal) and topological phase boundaries, leading to the development of novel tools for identifying topological phase transitions in non-Hermitian systems. Notably, the different response of the two-phase mechanisms to initial conditions and system parameters also opens promising avenues in quantum simulation and quantum sensing^19^. For example, selective detection of topological boundaries becomes achievable through precise manipulation of gain/loss ratios and evolution pathways, which thereby opens avenues for developing novel quantum sensors and topological quantum devices based on non-Hermitian effects. From a broader perspective, this work will promote the research of non-equilibrium quantum many-body systems into a new stage^20^, especially in the understanding of key issues such as the evolution of dissipative systems and topological protection mechanisms.
